# Fine-scale population genetic structure of the Bengal tiger (*Panthera tigris tigris*) in a human-dominated western Terai Arc Landscape, India

**DOI:** 10.1371/journal.pone.0174371

**Published:** 2017-04-26

**Authors:** Sujeet Kumar Singh, Jouni Aspi, Laura Kvist, Reeta Sharma, Puneet Pandey, Sudhanshu Mishra, Randeep Singh, Manoj Agrawal, Surendra Prakash Goyal

**Affiliations:** 1 Department of Ecology and Genetics, University of Oulu, Oulu, Finland; 2 Wildlife Institute of India, Chandrabani, Dehradun, India; 3 Population and Conservation Genetics, Instituto Gulbenkian de Ciência, Oeiras, Portugal; 4 Department of Wildlife Sciences, Amity University, Noida, India; University of Illinois at Urbana-Champaign, UNITED STATES

## Abstract

Despite massive global conservation strategies, tiger populations continued to decline until recently, mainly due to habitat loss, human-animal conflicts, and poaching. These factors are known to affect the genetic characteristics of tiger populations and decrease local effective population sizes. The Terai Arc Landscape (TAL) at the foothills of the Himalaya is one of the 42 source sites of tigers around the globe. Therefore, information on how landscape features and anthropogenic factors affect the fine-scale spatial genetic structure and variation of tigers in TAL is needed to develop proper management strategies for achieving long-term conservation goals. We document, for the first time, the genetic characteristics of this tiger population by genotyping 71 tiger samples using 13 microsatellite markers from the western region of TAL (WTAL) of 1800 km^2^. Specifically, we aimed to estimate the genetic variability, population structure, and gene flow. The microsatellite markers indicated that the levels of allelic diversity (*MNA* = 6.6) and genetic variation (*Ho = 0*.*50*, *H*_*E*_ = *0*.*64*) were slightly lower than those reported previously in other Bengal tiger populations. We observed moderate gene flow and significant genetic differentiation (*F*_*ST*_= 0.060) and identified the presence of cryptic genetic structure using Bayesian and non-Bayesian approaches. There was low and significantly asymmetric migration between the two main subpopulations of the Rajaji Tiger Reserve and the Corbett Tiger Reserve in WTAL. Sibship relationships indicated that the functionality of the corridor between these subpopulations may be retained if the quality of the habitat does not deteriorate. However, we found that gene flow is not adequate in view of changing land use matrices. We discuss the need to maintain connectivity by implementing the measures that have been suggested previously to minimize the level of human disturbance, including relocation of villages and industries, prevention of encroachment, and banning sand and boulder mining in the corridors.

## 1. Introduction

Knowledge of the genetic structure and gene flow in wild animal populations is crucial for making decisions to improve their sustainability. Factors known to influence the genetic structure and gene flow in animals include behavioral traits such as dispersal, social behavior and mating systems [1, 2], landscape features [3, 4, 5], availability of resources [6], and climate change [7]. Gene flow is often considered beneficial for maintaining local genetic variation as it counteracts the effects of genetic drift and spreads potentially adaptive alleles [8]. Conversely, gene flow might counteract local adaptations by importing maladaptive traits, by genetic swamping or by disrupting locally adaptive gene complexes [9]. Reduced gene flow between populations results in isolation and an isolated population may accrue significant genetic differences from other populations of the same species [10].

Large carnivores have the ability to go across long distances and endure in diverse environmental conditions [11, 12]. However, during the last two centuries, many large carnivore species have faced severe threats as their geographic ranges have contracted and habitats have fragmented [13, 14]. The loss of habitat will constrain movements, thus reducing population densities and sizes [15, 16]. Small populations always prone to demographic stochasticity, not just in a fragmented landscapes or small protected areas [17]. The shrinkage in geographic range and habitat fragmentation due to anthropogenic disturbances may also lead to human–carnivore conflict [18]. Ultimately, fragmentation and loss of habitat result in the isolation of populations, which in the long-term reduces genetic variation and increases extinction probability due to inbreeding and reduced fitness [19, 20]. The loss of genetic variability in many highly vagile and long-ranging species, such as Ethiopian wolves (*Canis simensis*) [21], pumas (*Puma concolor*) [22], Eurasian lynx (*Lynx lynx*) [23], brown bears (*Ursus arctos*) [24], jaguars (*Panthera onca*) [25], and Isle Royale wolves (*Canis lupus*) [26] has been affected by reduced movements of individuals together with other ecological/biological factors that have inhibited migrating individuals from contributing to gene pools. As genetic variability is often directly associated with the survival of individuals, knowledge of genetic variation and fine-scale spatial structuring is essential for endangered species, such as the tiger (*Panthera tigris*). In addition, knowledge of the patterns of gene flow is crucial for developing conservation plans by identifying population units and source populations that require management [19].

The tiger is an iconic species for the conservation initiatives because of its role as an apex predator in various ecosystems. All 13 tiger range countries have been experiencing profound economic growth over the last two decades [27], and as a consequence, urbanization and encroachment of habitats for extensive infrastructure development have imposed unprecedented pressures on tiger habitats [28, 29, 30]. India is home to about 70% of the global tiger population [31]. The Bengal tiger (*Panthera tigris tigris*) is found in six tiger landscape complexes in India [32]. Of these, the Terai Arc Landscape (TAL) of 42,700 km^2^, one of the 42 global source sites of tigers [33], is a unique habitat in the foothills of the Himalaya, notable for the richness of prey species for tigers [34, 35]. TAL has a denser human population (over 500 people/km^2^) than the national average in India (300 people/km^2^) [36], hence most of the tiger habitat in TAL has been encroached upon for development or increased agriculture production.

**Fig 1:**
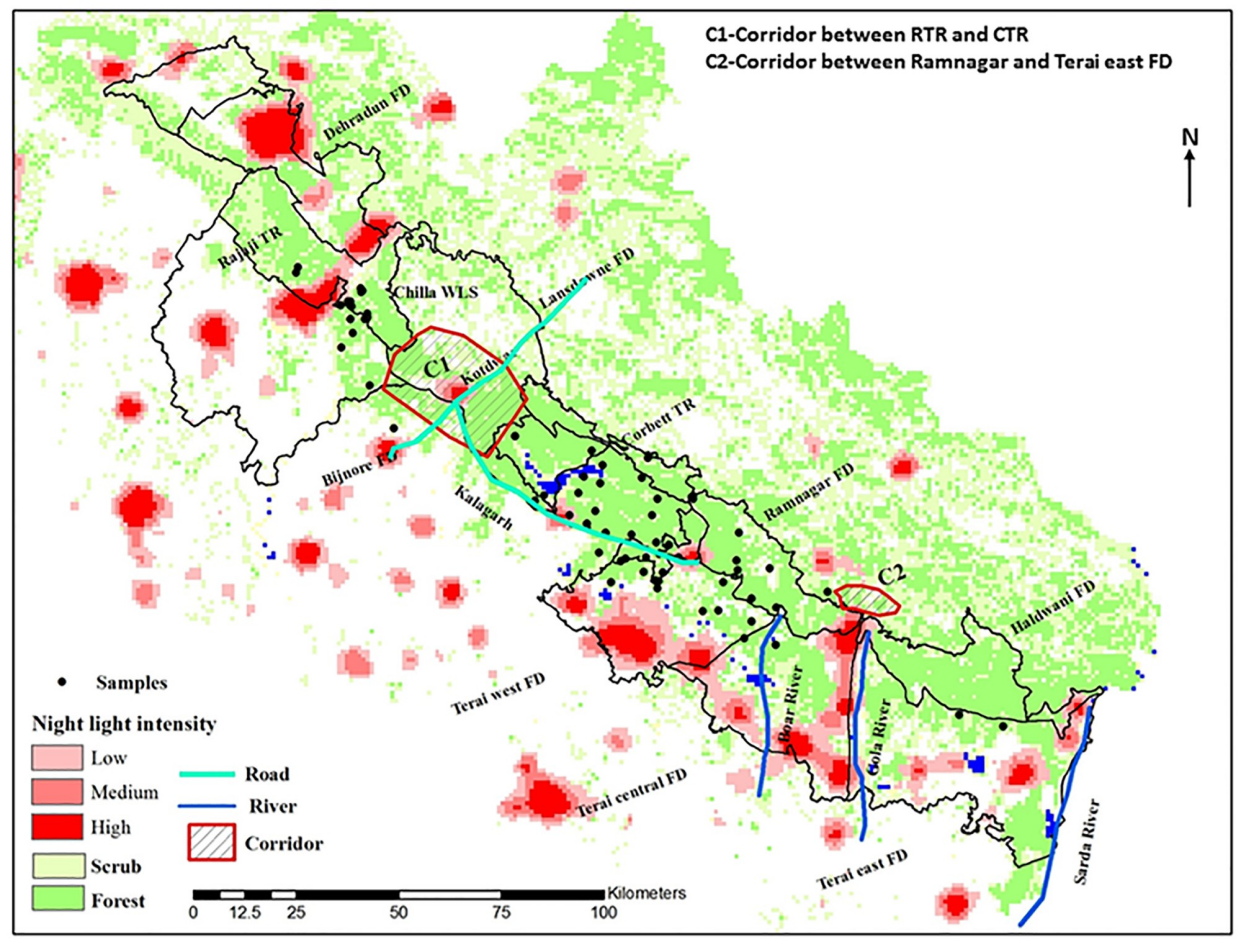
Map showing tiger samples locations collected from WTAL, human habitation depicted by the amount of night light pollution and identified corridors C1&C2.

The tiger is found in patchy habitats with forests, agricultural land, and human habitations in the TAL region of India [36]. The western region of TAL (WTAL) forms the northern distribution edge of the Bengal tiger in the Indian subcontinent [34]. In WTAL, there are two protected areas, the Rajaji Tiger Reserve (RTR) and the Corbett Tiger Reserve (CTR) (Fig 1). Of these two, CTR is the only source population of tigers in this area, and is responsible for maintaining genetic connectivity among the entire northwestern tiger population of TAL [32] and possibly for the central to eastern part of TAL as well.

Several ecological studies have emphasized the need to reduce anthropogenic pressure and restore corridors to provide a better opportunity for large mammals to move between the protected areas in WTAL [36, 37, 38, 39]. In RTR, loss of connectivity in the Chilla–Motichur corridor resulted in the extinction of the tigers from the western part of RTR [38] (Fig 1). Johnsingh and Negi [34] reported that tigers are rare also in the RTR–CTR corridor and have gone extinct in the four forest divisions of WTAL and suggested several management measures. Harihar et al. [37] and Harihar and Pandav [39] suggested many measures (e.g. relocation of villages in the corridor and protected areas) to reduce anthropogenic pressure and restore habitat.

Reduction of anthropogenic pressure has been shown to be effective in tiger management in this region. For example, the Gujjars, a pastoralist community in WTAL, were relocated from the eastern part of RTR, facilitating the recolonization of this habitat by tigers from the source population of CTR [37, 38]. Reducing the anthropogenic pressure and relocating human habitations from the tiger’s natural habitat will eventually curtail the risk of inbreeding, territorial fights, human–wildlife conflicts, and local extinctions. These have been the major conservation tactics of the National Tiger Conservation Authority of the Government of India to secure inviolate and crucial tiger habitats across India [40].

In the absence of ecological and biological details on movements of tigers, genetic characteristics can be used to understand the responses of species to the landscape and anthropogenic features. However, most genetic studies conducted on the Bengal tiger have been either in some way focused on the global status [41, 42] or were carried out in different tiger landscapes or regions, such as the Central and Peninsular [43, 44], Western (e.g. Ranthambhore Tiger Reserve) [45] and Sundarbans [46]. Despite the current knowledge of the tiger’s ecology, there is no information on the gene flow and genetic structure of the tiger population especially in WTAL, which holds the largest number of tigers [32], except from mitochondrial (mtDNA) markers [47,48]. Genetic variation in mtDNA was found to be low in the TAL tiger population and this population was found to be distinct from central Indian tigers due to the presence of a different mtDNA haplotype [47]. Within the TAL, there was some evidence for genetic isolation of the tigers west of the river Ganges, which is in the western part of the RTR, while the rest of the TAL was found to hold a uniform tiger population [48].

Therefore, we sampled tigers across most of the WTAL region and assessed the level of genetic connectivity and the population’s genetic structure in this globally significant tiger landscape. Given the previous ecological knowledge and the lack of genetic information, the present study focused on the following objectives: (1) genetic characterization and determination of population structure; (2) determination of the effect of anthropogenic pressure and recent developmental activity on genetic structure; (3) tracing the demographic history of the Bengal tiger in WTAL.

## 2. Material and methods

### 2.1. Ethics

All the blood, tissue and skin samples used in the present study were from tigers that had died naturally or in conflicts, and were provided by the Forest Department, Uttarakhand, to the national wildlife reference sample repository at the Wildlife Institute of India. Tiger scat samples were collected noninvasively without animal capture or handling. Therefore, sample collection did not require any handling of the animals. All necessary permissions to collect and store the samples at the national wildlife reference sample repository were obtained from the Ministry of Forest, Environment and Climate Change, Government of India and Forest Department, Uttarakhand (letter No. 1/29/2003-PT).

### 2.2. Study area

The study was carried out in WTAL, comprising an area of 1800 km^2^, including RTR (Rajaji Tiger Reserve), CTR (Corbett Tiger Reserve) and the adjoining forest areas, falling within the Bijnore, Lansdown, Ramnagar, Terai West, Central and Terai East forest divisions (FD) of TAL. There are several identified corridors (such as Rajaji-Corbett, Kosi river and Nihal Bhakra) connecting tiger reserves and FDs in WTAL (Fig 1).

### 2.3. Sample collection and extraction of DNA

A total of 71 tiger samples (59 tissue or blood samples, 12 scat samples) available at the Wildlife Institute of India were used. All tissue, blood and scat samples were collected between 2005 and 2014 across WTAL (RTR, n = 15; CTR, n = 27 and Terai west, central and east FDs, n = 15 and Ramnagar FDs, n = 14) by the Forest Department of Uttarakhand. Scat samples (*n* = 12; collected in 2009) were used previously in a study by Singh et al. [46]. Extractions of DNA from the scat and tissue/blood samples were carried out using QIAamp DNA Stool and DNeasy Blood and Tissue Kit (QIAGEN, Germany), respectively, and all necessary precautions were taken to avoid contamination.

### 2.4. Genetic analyses

#### 2.4.1. Amplification of microsatellite markers and genotyping

Thirteen highly polymorphic fluorescently labeled microsatellite loci, namely PttA2, PttD5, PttE5 and PttF4 [49], PUN100 and PUN327 [50] and FCA304, FCA272, F41, FAC672, FCA232 FCA126 and FCA090) [51] were amplified. In each reaction, the total volume was 10 μl, with 1 μl of 50x dilution of the extracted DNA, 5 μl of the 1× multiplex PCR Master Mix buffer (QIAGEN Multiplex PCR Kit, Germany), 1× of BSA and 0.4 μM of each primer. The thermal profile of the amplification was as follows: initial denaturation at 94°C for 15 minutes, followed by 40 cycles of denaturation at 94°C for 35 seconds, annealing at 55°C [49], 53°C [50] and 51°C [51] for 1 minute and extension at 72°C for 90 seconds, with one cycle of final extension for 30 minutes at 72°C. The amplified PCR product was subjected to fragment analysis on an ABI 3130 Genetic Analyzer (Applied Biosystems). The alleles were scored with Gene Mapper 3.7 (Applied Biosystems).

#### 2.4.2. Genotyping error and data validation

About 20% (12 of 59) of the field-collected tissue and 60% (7 of 12) scat samples from WTAL were used to address genotyping error. Each sample was genotyped three times, and the maximum likelihood of allele dropout (ADO) and false allele (FA) error rates were quantified using PEDANT version 1.0 with 10,000 search steps for enumerating each error rate [52]. The scoring errors were assessed and validated using MICROCHECKER 2.2.2 [53]. We rounded all genotype calls either even or odd numbers for respective loci, and most of the differences between the assigned and actual allele sizes were between 0.3 bp and 0.5 bp.

#### 2.4.3. Genetic diversity and assessment of inbreeding

Genetic diversity measures, expected heterozygosity (*H*_*E*_), observed heterozygosity (*H*_*O*_), mean number of alleles, and allelic richness (*A*_*R*_) were estimated using FSTAT [54]. The Hardy–Weinberg Equilibrium (*HWE*) was checked with the null hypothesis of random union of gametes at each locus within and across sampling locations at each study area using the exact test [55] in GENEPOP [56]. Bonferroni correction was applied for multiple comparisons. The linkage disequilibrium (*LD*) among all the locus pairs was also calculated using GENEPOP [56]. Wright’s inbreeding coefficient (*F*_*IS*_) was estimated for the whole WTAL and for the two sampling sites (RTR and CTR) according to the method of Weir and Cockerham [57] using GENEPOP [56].

#### 2.4.4. Effective population size and demographic history

The effective population size (*N*_*E*_) was calculated using an approach based on the *LD* as performed in LDNe 1.31 [58]. The criterion for *Pcrit* was set to 0.02, which provides a balance between precision and bias from rare alleles [58]. A departure from the heterozygosity expected from the observed number of alleles under the assumption of mutation-drift equilibrium in the microsatellite data was tested using BOTTLENECK v.1.2.0.2 [59, 60]. Significant deviations can be due to changes in population sizes such as expansions and bottlenecks, assuming that the samples were obtained from a random mating and isolated population. The bottlenecked populations will show an excess of heterozygosity compared with that expected in equilibrium from the observed allelic diversity. BOTTLENECK program was run under two mutation models: two-phased (TPM), and stepwise mutation (SMM). The TPM was set at 95% stepwise mutation, 5% multi-step mutations, as recommended by Piry et al. [60]. Variance was 12 for the TPM. Wilcoxon signed-rank tests were used to identify the heterozygosity excess [60].

A bottleneck in a population may induce a distortion in size distribution of microsatellite alleles [59]. This gap in distribution can be quantified by the Garza-Williamson index (G-W), the mean ratio of the numbers of observed alleles to all the potential repeats within the allele size range, across all loci [61,62]. Therefore we estimated the G-W index [61] with Arlequin 3.5.2.1. [62]

#### 2.4.5. Population genetic structure

Bayesian clustering and non-Bayesian multivariate analyses were used to detect genetic structure. Several individual Bayesian clustering-based programs were used. In some of these programs, individuals were assigned exclusively on the basis of their multilocus genotypes (e.g. STRUCTURE), while others used both multilocus genotype and geo-referenced information (e.g. TESS and GENELAND). Multivariate ordination analyses, such as the discriminant analysis of principle component (DAPC) and spatial principle component analysis (sPCA) were also used, as these can provide a useful validation of Bayesian clustering output [63, 64] not being based on any model assumptions.

STRUCTURE [65] uses a Bayesian-based Markov chain Monte Carlo (MCMC) approach and was used to propose the number of populations (K) in the data. The number of populations (K) was inferred using an admixture model, and the allele frequencies were considered correlated. The Bayesian clustering analyses were carried out both with (*LOCPRIOR* = 1) and without prior (*LOCPRIOR* = 0) knowledge of sampling locations. A series of 20 independent runs was conducted for each value of K between 1 and 10, with a burn-in period of 50,000 iterations and data were collected for 500,000 iterations. STRUCTURE was run using a data set comprising samples from (1) WTAL, including RTR, CTR and the adjoining FDs and (2) CTR and the adjoining FDs. Using the posterior probabilities of the data for a given K (ln P (K)) and the second-order rate of change of the log probability of the data between consecutive values of ΔK [66], calculated in the program STRUCTURE HARVESTER v.0.6.8 [67], the most likely K values were selected. We considered a membership coefficient (*q*) above 0.7 as a realistic cut-off value to assign an individual to a population.

TESS v.2.3 [68] was run using the conditional autoregressive admixture model with the spatial interaction parameter set at 0.6, as recommended by Chen et al [68]. One hundred replicate runs of 100,000 sweeps (disregarding the first 30,000) were performed for K values from 1 to 10. The preferred K was selected by comparing the individual assignment results and the deviance information criterion (DIC) for each K [69]. DIC values, averaged over 100 independent iterations, were plotted against the K values, and the most likely value of K was selected by visually assessing the point at which DIC first reached a plateau. GENELAND v.4.0.3 [70] was run through an extension of R v.3.0.1 under the correlated allele frequency model without spatial uncertainty in spatial locations. K was allowed to vary between 1 and 10 in 20 independent runs, each with 105 iterations, with thinning set to 100, the maximum number of nuclei to 1000 and the maximum rate of the poisson process to 333.

The presence of null alleles may bias the results obtained with the Bayesian approach because of deviations from the HWE. Multivariate analyses constitute an alternative approach and can be used to validate individual Bayesian clustering. Discriminant analysis of principal components (DAPC), a non-model-based method that has been developed recently and implemented in the adegenet R package [71], provides an efficient description of genetic clusters using a few synthetic variables, called discriminant functions. This analysis seeks linear combinations of the original variables (alleles) that show differences between groups, while minimizing variations within clusters. DAPC does not require a population to be in HWE and linkage equilibrium (LE). Spatial principle component analysis [71] was also used, as it can categorize cryptic spatial patterns of genetic structure across a landscape, including clines, by accounting for spatial autocorrelation related with neighbor-mating and sample distribution. In WTAL, the distribution of tigers is contiguous, following a stepping-stone model on a large spatial scale. To allow visualization of the pattern of the genetic distance (diversity) across the landscape, the Alleles In Space (AIS) software package [72] was used, with a 50×50 grid surface and distance weighting parameter set at 1, to obtain a genetic landscape interpolation (GLSI) plot.

#### 2.4.6. Gene flow and migration rate

The pairwise *F*_*ST*_ was used as an indirect measure to examine the historical gene flow. It was calculated using Arlequin 3.5.2.1 (*p = 0*.*05*, 10,000 randomization) [62]. This package [62] was also used to estimate the proportions of the total genetic variance arising from within and between populations, using analysis of hierarchical molecular variance (AMOVA). We grouped individuals into two groups, with RTR in one group and CTR and adjoining FDs in another.

Further, three more analyses, i.e. the likelihood-based estimator, posterior probability distribution, and sibship analysis, were used to identify migrants between RTR and the source population (CTR) and residents. We categorized as a migrant an individual that does not originate from the sampled population and a resident (non-migrant) as an individual that originates from the sampled population. The recent migration (over the last 5–6 generations) was calculated using a Bayesian MCMC method implemented in BAYESASSv.1.3 [73]. The method accounts for deviations from HWE within populations by incorporating a separate inbreeding coefficient for each population. The program was run for 3×10^−7^ iterations, of which 1×10^−7^ was discarded as a burn-in. Multiple runs were carried out with different seed numbers and delta values to ascertain the final parameter that would accept 40–60% of changes in the total chain length and to examine convergence and consistency among runs.

A likelihood-based estimator was also used to identify migrant, admixed and resident individuals. Exclusion probabilities were calculated using the Monte Carlo method of Paetkau et al. [74], because this approach is considered to be less prone in excluding resident individuals erroneously compared with other methods. Migrants in each population were identified using the Bayesian criterion of Rannala and Mountain [75] and the re-sampling method of Paetkau et al. [74] as implemented in GENECLASS 2.0 [76] to determine the critical value of the test statistic (*Lh* or *Lh/Lmax)* beyond which individuals could be assumed to be migrants [77]. An alpha value of 0.01 was used to determine the critical values [75].

Sibship analysis is a novel approach in detecting migrants, admixed individuals and residents in a population and has been successfully used previously, for example for wolves [78]. Waples and Gaggiotti [79] emphasized that sibship analyses using multilocus genotyping data can provide direct and indirect estimates of migration and reveal the fine genetic structure within populations. The COLONY software package [80, 81] was used to carry out sibship analysis with the full likelihood approach. A standard frequency of null alleles and genotyping error rate (0.05) were used. The same criteria that were previously used for wolves [78, 82] were used to categorize migrant and resident individuals: the presence of between-population sibship would indicate migration events between populations, while sibship within the sampled locality would indicate residents.

#### 2.4.7. Prey density and level of anthropogenic disturbance in WTAL

The distribution and movements of tigers are largely governed by the availability of prey [83], the level of disturbance in the habitats [31], and the corridors connecting adjoining populations [84]. Therefore, to correlate the observed population genetic structure in WTAL with the prey distribution and disturbance level, the available literature was examined and compiled for different FDs as well as protected areas [31, 34, 36, 39, 85]. Human habitation and town development were depicted in the form of night light pollution using Geographic Information System (GIS). We used the data available from the United States Defense Meteorological Satellite Program and the National Oceanic and Atmospheric Administration’s Operational Line scan System (http://www.ngdc.noaa.gov/dmsp/sensors/ols.html; accessed 23 August 2011) and analyzed it in ArcGIS 10.0 (ESRI 2011) software.

## 3. Results

### 3.1. Error rate

We did not observe any allelic dropout or false alleles in the tested tissue samples, which comprised 20% (n = 12) of all tissue samples. Within the scat samples, genotyping error rate varied among loci, however, ADO rate ranged from 0% to 17% and FA between 0 to 5% (S1 Table). The mean allele dropout rate was 5%, which is comparable to the reported error rate in other studies [86, 87]. Null allele frequency ranged from 0.01 to 0.27 across the loci. Among the 13 loci, four (PttE5, PttF4, PUN100 and FCA090) showed a frequency of more than 15%. We also checked for null alleles within individual sampling locations from WTAL tiger populations and found indications that several loci have null alleles, but this was not consistent over the different sampling sites (Table 1).

**Table 1:** Genetic characterisation of tiger in WTAL, India.

Locus	CTR**							RTR							Overall						
	*N*	*A*	*A*_*R*_	*H*_*O*_	*H*_*E*_	*F*_*IS*_	*F*_*null*_	*N*	*A*	*A*_*R*_	*H*_*O*_	*H*_*E*_	*F*_*IS*_	*F*_*null*_	*N*	*A*	*A*_*R*_	*H*_*O*_	*H*_*E*_	*F*_*IS*_	*F*_*null*_
**PttA2**	54	6.0	4.37	0.46	0.63	0.28	0.16	15	3.0	2.80	0.60	0.48	-0.20	-0.11	69	6.0	5.85	0.49	0.61	0.20	0.11
**PttE5**	55	4.0	3.53	0.27	0.42	0.37*	0.22	15	6.0	5.73	0.46	0.74	0.40	0.22	70	6.0	5.83	0.31	0.57	0.45	0.27
**PttF4**	51	4.0	2.79	0.37	0.53	0.31*	0.18	15	3.0	3.00	0.20	0.58	0.67*	0.47	66	4.0	3.90	0.33	0.56	0.41*	0.24
**PttD5**	56	7.0	4.73	0.66	0.69	0.05*	0.02	14	4.0	3.96	0.14	0.62	0.78*	0.61	70	7.0	6.97	0.55	0.70	0.21	0.10
**FCA304**	55	5.0	3.74	0.63	0.67	0.06	0.03	15	4.0	3.80	0.53	0.67	0.24	0.11	70	6.0	5.71	0.61	0.67	0.09	0.05
**FCA272**	53	5.0	4.84	0.69	0.75	0.08	0.04	14	6.0	5.71	0.50	0.65	0.27	0.14	67	7.0	6.89	0.65	0.76	0.15	0.07
**F41**	52	6.0	4.07	0.48	0.49	0.04	0.02	14	3.0	2.98	0.28	0.30	0.11	0.09	66	5.0	4.99	0.42	0.46	0.09	0.03
**PUN327**	56	7.0	4.79	0.53	0.58	0.09	0.04	15	7.0	6.95	0.33	0.80	0.61*	0.40	71	8.0	7.82	0.49	0.69	0.29	0.15
**PUN100**	56	5.0	4.44	0.44	0.65	0.32*	0.19	12	5.0	5.00	0.41	0.69	0.43*	0.26	68	6.0	5.99	0.44	0.72	0.39*	0.23
**FCA126**	47	7.0	4.92	0.55	0.65	0.16	0.08	13	5.0	4.84	0.38	0.63	0.42	0.23	60	10.0	10	0.51	0.67	0.24	0.14
**FCA672**	54	7.0	5.00	0.70	0.68	-0.02*	-0.03	15	7.0	6.36	0.93	0.69	-0.31	-0.17	69	9.0	8.60	0.75	0.69	-0.07	0.05
**FCA232**	50	5.0	0.12	0.44	0.49	0.12	0.06	14	2.0	2.00	0.42	0.33	-0.23*	-0.11	64	5.0	4.87	0.43	0.46	0.06	0.01
**FCA090**	50	6.0	0.73	0.40	0.73	0.46*	0.31	15	7.0	6.95	0.60	0.83	0.31*	0.15	65	8.0	7.98	0.44	0.77	0.42*	0.27
**Mean**		**5.69**	**4.28**	**0.51**	**0.61**	**0.18**	**-**		**4.7**	**4.62**	**0.44**	**0.62**	**0.31**	**-**	**67**	**6.69**	**6.57**	**0.50**	**0.64**	**0.23**	**-**

*N*, number of sample used; *A*, number of allele; *A*_*R*_, allele richness; *H*_*o*_, observed heterozygosity; *H*_*E*_, expected heterozygosity; *F*_*IS*_, inbreeding coefficient; *F*_*null*_, null allele frequency.

* *P*<0.05

** includes Corbett Tiger Reserve (CTR), Forest Divisions of Lansdown, Ramnagar, Terai West and Terai Central.

RTR-Rajaji Tiger Reserve.

### 3.2. Genetic diversity, departure from HWE

All the markers (n = 13) were polymorphic, with 4–10 alleles (Table 1). The basic genetic diversity values, measured by the mean number of alleles (*MNA*) and allelic richness (*A*_*R*_), were 6.69 and 6.57, respectively. The observed and expected heterozygosity in WTAL were 0.50 and 0.64, respectively. Deviations from HWE were inferred for five loci (PttE5, PttF4, PttD5, PUN100 and FCA090) in CTR and in six loci (PttF4, PttD5, PUN327, PUN100, FCA232 and FCA090) in the RTR tiger populations. When the data were tested globally, it was found that only three loci (PttF4, PUN100 and FCA090) were consistently showing deviations across the sampling sites. These three loci also showed the high frequency of the null allele (PttF4, PUN100, FCA090; Table 1) across the sampling site (populations). Therefore, consistent HWE deviation in these three loci might be due to the null allele, and it can bias the several genetic analysis. Hence, these three loci were excluded from subsequent analysis. No significant linkage disequilibrium was found. The mean inbreeding coefficient (*F*_*IS*_) was significantly different from zero and was positive for both populations (0.18 for CTR and 0.31 for RTR) and the overall value was 0.23 (Table 1).

### 3.3. Effective population size and demographic history

The effective population size was estimated to be 81.2 (95% CI: 47.7–195.9) for the CTR and 46.8 (13.6 to infinite) for RTR populations (Table 2). The results of the BOTTLENECK analysis showed that there was no consistent or strong signal for a departure of heterozygosity from mutation drift equilibrium (Table 2). Also, all allele frequency distributions were typical (L-shaped) to non-bottlenecked populations in WTAL. Garza and Williamson [61], suggested that values of the *G-W* index lower than 0.68 is evidence of a bottleneck, whereas values greater than 0.68 would denote no bottleneck history. In the present data set, the *G-W* values were between 0.73 and 0.77 (Table 2).

**Table 2:** Summary of bottleneck analyses and effective population sizes (N_E_) in WTAL, India.

Pop	Mutation model	Sign test	Standardized difference test	Wilcoxon test (H deficiency/H excess/H excess & deficiency	Allele frequency distribution	M-ratio (G-W index)	*N*_*E*_ (CI 95%) (Pcrit = 0.02)
CTR*	TPM	*p* = 0.065	*p* = 0.385	*p* = 0.278/0.996/0.556	L-shaped	0.768	81.2(47.7–195.9)
	SMM	*p* = 0.013^#^	*p* = 0.0001^#^	*p* = 0.004^#^/0.996/0.009#			
RTR	TPM	*p* = 0.580	*p* = 0.393	*p* = 0.577/0.460/0.921	L-shaped	0.726	46.8 (13.6- infinite)
	SMM	*p* = 0.407	*p* = 0.116	*p* = 0.246/0.784/0.492			

Pop = population, TPM = two phase mutation model, SMM = stepwise mutation model

* includes Corbett Tiger Reserve (CTR) and Forest Divisions of Lansdown, Ramnagar, Terai West and Terai Central.

RTR—Rajaji Tiger Reserve.

^#^significant p-value (p<0.05)

### 3.4. Population structure, gene flow and migration rate

**Fig 2:**
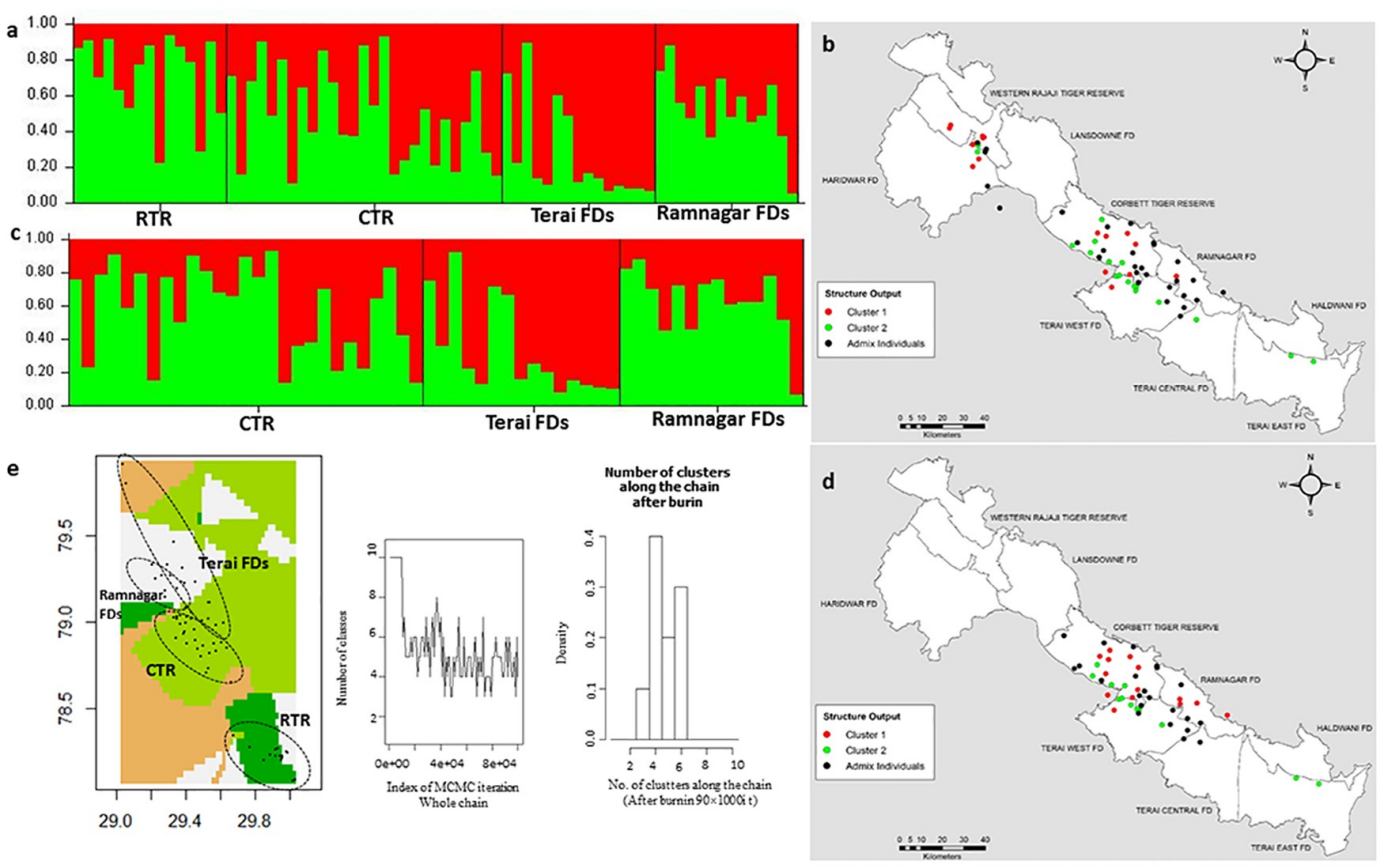
Bayesian clustering analysis result using genetic programs STRUCTURE and GENELAND based on 10 microsatellite loci and admixture model. a) Summary bar plot of STRUCTURE run at K = 2 showing population assignments for each individual of the populations from western TAL including RTR, CTR and adjoining Forest Division b), Map showing individuals by dots and colored to reflect the cluster they were assigned to with the highest probability (>70%) in 2a. Black color represents admix individual (<70%), c). Summary bar plot of STRUCTURE run at K = 2 showing population assignments for each individual of the populations from CTR and adjoining Forest Divisions, d) Map showing individuals by dots and colored to reflect the cluster they were assigned to with the highest probability (>70) in 2c. Black color represents admix individual (<70%), d) GENELAND analysis result. Colors in figure indicate different populations in WTAL and dots show sampled locations of individuals.

The Bayesian cluster analysis with STRUCTURE indicated two as the most probable number of genetic clusters (K = 2), by the mean likelihood (mean ln P (k) = -1267) [65] and Delta K value [66]. Both with and without prior knowledge of sampling locations yielded two population clusters, but the clustering pattern was slightly stricter with the locprior than without. Without the locprior model it was found that both populations shared ancestry and only 50% of the individuals were completely assigned (q>0.7), while 50% of the individuals showed mixed ancestry (q<0.7) (Fig 2a and 2b). However, using the locprior model all of the individuals were completely assigned to their respective sampling locations, except for one individual sampled from Rajaji that showed proximity to the CTR population (see S1 Fig). In the separate analysis of only CTR and the adjoining FDs, we found a similar clustering (Fig 2c and 2d) pattern and detected that individual from the west; central and east FDs in WTAL formed a separate cluster, while individuals from the CTR and Ramnagar were assigned to another cluster.

Population clustering analysis with spatial information implemented in TESS gave results similar to that of STRUCTURE with locprior information and suggested that there are two clusters in WTAL, with a few individuals migrating between these (see S2 Fig). GENELAND inferred four clusters (k = 4), but most of the individuals were assigned only to three clusters with high spatial consistency and clearly defined cluster boundaries (Fig 2e), and only four individuals were assigned to the fourth cluster.

**Fig 3:**
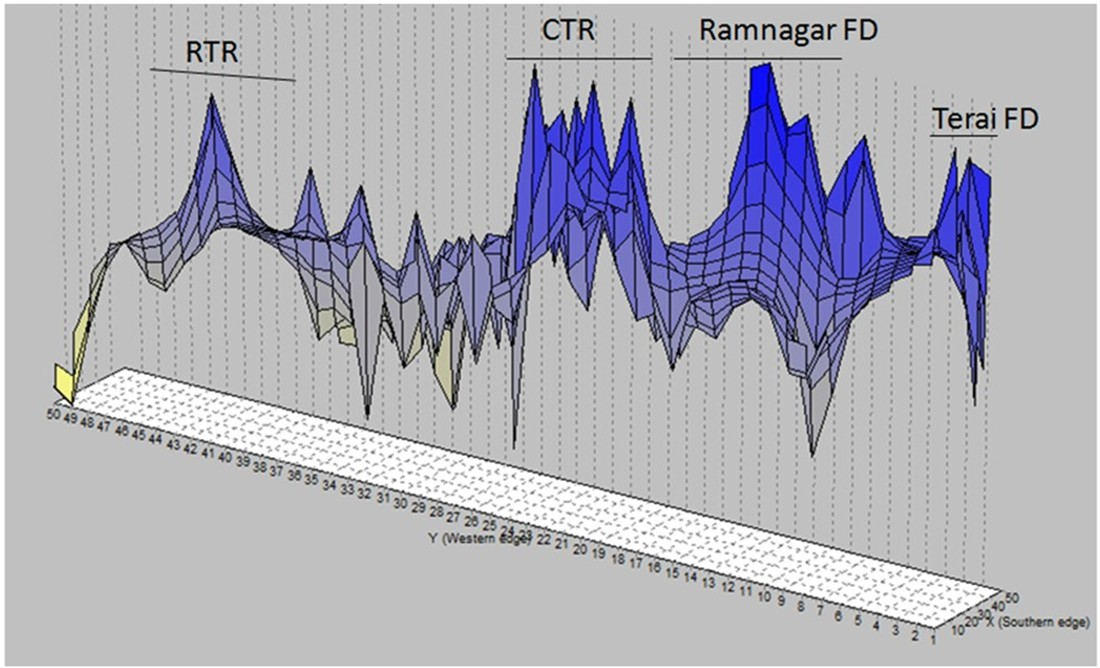
Genetic Landscape Shape Interpolation (GLSI). Surface plot heights reflect genetic distance patterns over the geographical landscape examined.

Taken as a whole, the different Bayesian clustering methods converged in identifying two distinct genetic clusters, i.e. RTR and CTR including adjoining FDs. The third cluster detected by GENELAND supports the subdivision of CTR and its adjoining FDs also identified by STRUCTURE (Fig 2c). The multivariate DAPC identified K = 3 or 4 as the optimal number of clusters according to the Bayesian information criteria (see S3a Fig). The DAPC output supports the findings of the other Bayesian clustering methods (STRUCTURE, TESS and GENELAND) that there are at least two populations in WTAL. The subdivision in CTR and adjoining FDs identified by STRUCTURE and GENELAND was not as clear in the DAPC analysis, as the spatial extant of the clusters of CTR and its adjoining FDs (see S3b Fig) overlapped substantially although the centroids were separate. sPCA (see S4 Fig) and GLSI from AIS (Fig 3) revealed variation in allele frequency from western RTR to CTR and its adjoining forest divisions. However, shade gradients in sPCA quantify the degree of genetic differentiation and the observed large value also reveals that the tigers from WTAL form two genetic clusters (see S4 Fig).

The pairwise *F*_*ST*_ value (*F*_*ST*_ = 0.060, *p* = 0.01) between RTR and CTR tiger populations in WTAL suggested moderate historical gene flow among the populations. The level of genetic differentiation in CTR and adjoining forest divisions suggested high gene flow between these populations (*F*_*ST*_ values ranged 0.01 to 0.04, Table 3). AMOVA analyses revealed that most of the variance (93.14%) originates from within the populations while variance between populations and among group is less, i.e., 1.56% and 4.54% respectively. The *F*_*ST*_ value calculated with AMOVA (*F*_*ST*_ = 0.061; p<0.001) also suggests significant differentiation between the populations (S2 Table). The long-term migration using BAYESASS resulted in low and asymmetric migration rates, more from CTR to RTR (0.089%) than from RTR to CTR (0.007%) (Table 4). GENECLASS 2.0 detected only two first-generation migrants from CTR to RTR and one that had migrated from RTR to CTR (S3 Table).

**Table 3:** Pairwise *F*_*ST*_ between the populations in the WTAL, India.

	CTR	Terai West, Central and East FDs	Ramnagar FD	RTR
CTR	0			
Terai West, Central and East FDs	0.016			
Ramnagar FD	0.041*	0.033*		
RTR	0.060*	0.11*	0.093*	0

*P<0.05

FD: Forest Division

CTR: Corbett Tiger Reserve

RTR: Rajaji Tiger Reserve

**Table 4:** Migration rates detected using BAYESASS for each population with 95% credible set.

	From	
Into	CTR*	RTR
CTR*	0.9921±0.007 (0.972, 0.999)	0.0078±0.007 (0.0002, 0.0276)
RTR	0.0893±0.042 (0.017,0.177)	0.9106±0.042 (0.822, 0.982)

*includes Corbett Tiger Reserve (CTR) and Forest Divisions of Lansdown, Ramnagar, Terai West and Terai Central.

**Fig 4:**
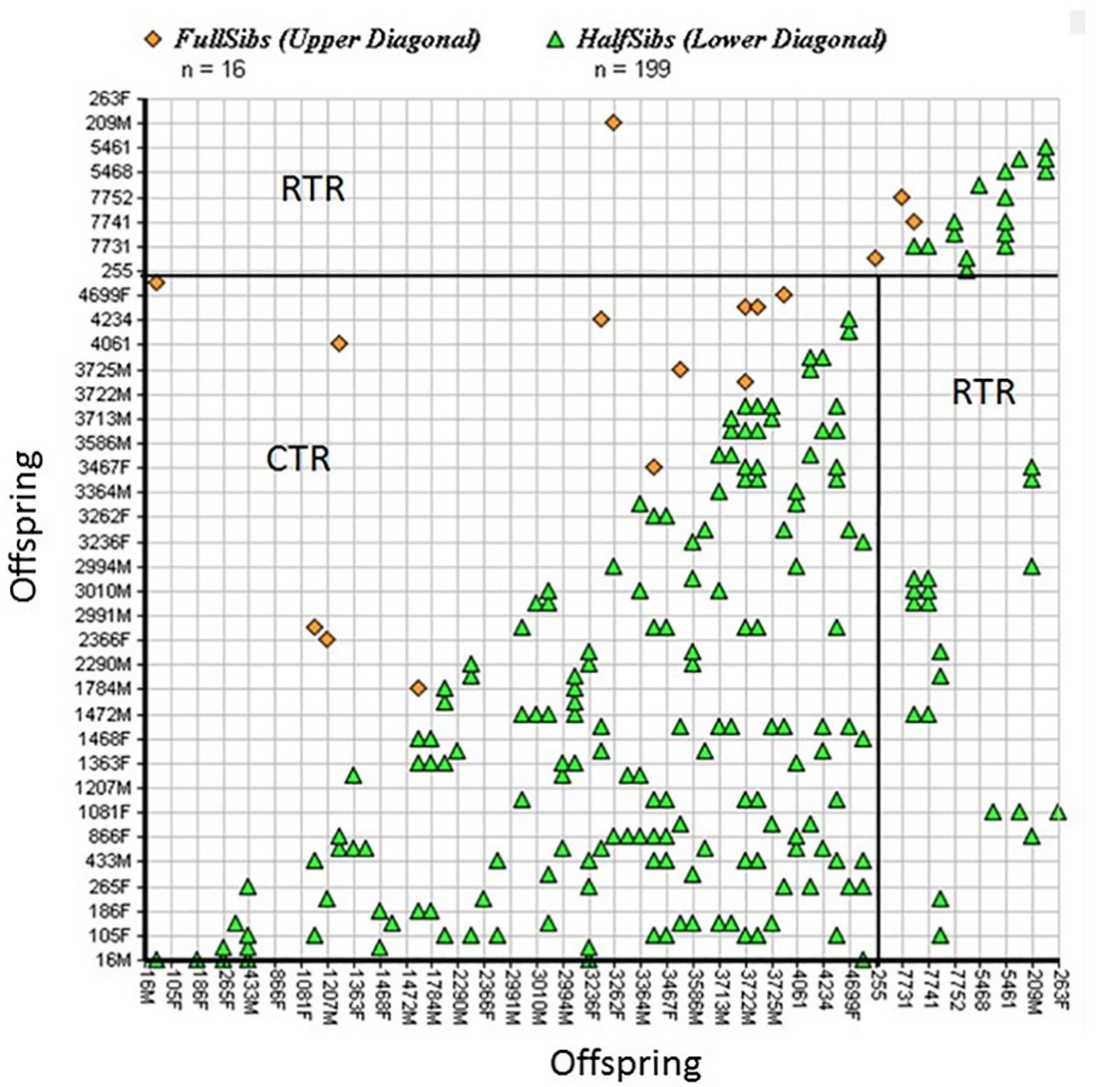
Sibship assignment using the program COLONY. Individuals are ordered such that members from the same population have consecutive indexes as listed on both x and y axes. RTR (Rajaji Tiger reserve; CTR (Corbett Tiger Reserve).

The results of the sibship assignment analysis carried out using COLONY are shown in Fig 4. Shared sibship between tigers from different populations is an indication of migration, while the frequency of siblings within a population indicates the effective population size and number of residents. There were more full and half sibship assignments within populations than between populations (Fig 4). The existence of a within-population sibship suggests that most of the individuals in Corbett and Rajaji are residents, but the existence of between-population sibship indicates migration of tigers between the populations. Overall, the number of half sibship is greater than the number of full sibship. CTR and its adjoining FDs have more sibship than RTR (Fig 4).

### 3.5. Prey density and disturbance factors

Quantitative assessment of distribution of wild prey species and their abundance (frequency of occurrence i.e. number of segments with presence signs/total segments sampled), habitat quality characteristics (wildlife dung density, percent canopy cover and tree density), and the extent of anthropogenic factors (dung and sightings of livestock i.e. cattle and buffalo, tracks and sightings of domestic dogs and people, lopping and cutting signs) showed that WTAL had no large scale differences in the habitat quality and abundance of prey species but protected areas are relatively better in these aspects than other FDs (Table 5). However, the population west of CTR experiences a comparatively high level of anthropogenic disturbance compared with that in the east (Table 5) [36]. Intensity of night light pollution is high in the Rajaji–Corbett corridor due to development of towns in Kotdwar (Fig 1), which was also reported by Qureshi et al. [88]. Harihar and Pandav [39] also reported that the Rajaji–Corbett corridor experiences more anthropogenic disturbance compared with the Kosi river corridor, in the eastern part of CTR.

**Table 5:** Wild prey species, habitat quality characteristics and extent of anthropogenic factors within the study area. (Values are means ±standard deviation).

Characteristics of the landscape	Eastern RTR (Chilla)	Rajaji–Corbett corridor, Bijnore and Lansdown FDs	Corbett Tiger Reserve	Ramnagar Forest Division	Terai West Forest Division	Terai Central Forest Division	Terai East Forest Division
*Percent frequency of occurrence of tiger (number of segments with carnivore signs / total number of segments surveyed)*							
Bengal Tiger (*Panthera tigris tigris*)	12.9±17.6	22.6±8.4 to 16.8±28.3	41.2±22.0	20.7±18.0	14.1±9.0	9.7±16.7	10.1±15.2
*Percent frequency of occurrence of major prey species (number of segments with prey / total number of segments surveyed)*							
Sambar (Rusa unicolor)	90.6±10.5	39.3±55.6 to 55±41.5	93.6±7.8	80±22.1	44±38	22±33	25.8±35.3
Chital (Axix axix)	88.3±19.8	100±0.0 to 28±36.6	80±22.0	58±36.5	75±32.8	29±31.5	48±37.4
Wild pig (Sus scrofa)	42±29.8	32±45.5to 23±33.1	24.7±19.9	36±30.7	65±21.5	33±(39)	42.5±39.1
Nilgai(Boselaphus tragocamaelus)	4.5±11.5	75±35.4 to 1.5±4.6	0	19.4±28.5	63±35.5	80±25.7	31±37.7
*Habitat characteristics*							
Wildlife dung density (number/ha)	2.4±0.3	2.5±2.4	1.3±1.6	0.5±0.08	0.3 ±0.6	0.9±1.5	0.2±0.5
Percent canopy cover	23.4±12.3	14.7±9.0	21.6±12.3	24.5±10.1	21.1±5.6	30.8±16.7	7.7±5.9
Tree density (number/ha)	8±4.4	6.5±3.7	7 ±5.7	9.4±4.2	13.8±4.1	13.8±6.2	20.8±12.6
*Anthropogenic parameters*							
Human encounter rate (number/km)	100	791	-	101	148	-	40
Looping (number/ha	1.5±2.4	0.8±1.3	0±0.01	0.1±0.4	0.1±0.5	0.9± 1.8	0.7±1.2
Livestock dung density (number/ha)	1.3±1.6	3.9±3.0	0.2±0.6	1.0±1.2	0.4 ±0.8	1.2± 1.2	1.9±2.

Sources: Johnsingh et al (2004).

- = Data not available

## 4. Discussion

### 4.1. Genetic diversity and demographic history

This study revealed the existence of a moderate level of allelic diversity and genetic variation (*H*_*O*_ = 0.50, *H*_*E*_ = 0.64; *A*_*R*_ = 6.5) in tiger populations from WTAL. However, all of these estimates are lower than those reported in Bengal tiger populations from other regions (*H*_*O*_ = *0*.*65 to 0*.*71*, *H*_*E* =_ *0*.*74 to0*.*81*, *A*_*R*_ = *7*.*76*) in India [42, 89], though, they are not directly comparable with the present study because of different markers used. The lower level of genetic variation in WTAL compared to other Bengal tiger populations might be due to the location of the populations at the northern limit of the tiger distribution range and due to a stepping-stone type of migration. Populations at the edge of the distribution range of a species often have lower genetic diversity as suggested by “the rear-edge” [90] or “abundant-center” hypotheses [91]. Mammals having demographically challenged populations history, often exhibit lower heterozygosity (*H*_*E*_ = 0.502±0.027) in comparison with stable, viable populations (*H*_*E*_ = 0.677±0.012) [92]. However, the disruption of gene flow between populations due to recent (in the last century) anthropogenic activities in this region is most likely responsible for the observed genetic erosion, and inbreeding (*F*_IS_) has probably already affected the population. Philopatric felids that are threatened by poaching are known to have high *F*_*IS*_ value as have been reported by other studies in viz tiger (*Panthera tigris tigris*) [43, 46], African leopard (*Panthera pardus pardus)* [93], African lion (*Panthera leo*) [94], puma (*Puma concolor)* [95], and ocelots (*Leopardus pardalis*) [96]. Recent habitat loss and forest fragmentation have reduced genetic diversity and increased genetic differentiation within the Bengal tiger populations in other regions [44, 46, 97]. Across the globe, large carnivores face similar threats and experience massive declines in their population sizes and geographic ranges [98], which may have resulted in low levels of genetic variation [19]. However, the present study did not find any signatures of a recent demographic bottleneck in the WTAL population but the genetic methods used do not always detect recent population declines [99]. Still, future increases in genetic drift within the WTAL population will probably lead to a more pronounced loss of genetic diversity.

### 4.2. Population structure, gene flow and migration rate

We found evidence that the tigers in WTAL are genetically structured forming at least two populations using both Bayesian and non-Bayesian methods. The tigers disperse over long distances, and forest corridors facilitate the movements between subpopulations and thus are important for maintaining the meta-population or for population sinks. WTAL is a good quality habitat for tigers due to the high prey density and presence of moderate forest cover [36]; however, recent ecological studies (Table 5) show a high disturbance within WTAL; for example, because of night light in this landscape (Fig 1). The present study supports the assumption that recent (in the last century) population fragmentation and increased urbanization have an impact on the genetic structure of the Bengal tiger in WTAL. The significant genetic differentiation and moderate gene flow (*F*_*ST*_ = 0.060) between CTR and RTR provide evidence that the Rajaji–Corbett corridor is more affected by anthropogenic disturbance in comparison to Kosi River and Nihal Bhakra corridors. The significant differentiation and high gene flow between CTR and Ramnagar FD (i.e. *F*_*ST*_ = 0.04) suggest that the corridor between these populations is functional. There is no differentiation (*F*_*ST*_ = 0.01) between CTR and the Terai East, Central and West FDs, indicating that there is sufficient migration and gene flow. Most of the eastern parts of CTR experience relatively low disturbance compared to the western part of CTR (Table 5), hence, the eastern part of CTR is considered as a contiguous tiger habitat and has maintained a high rate of gene flow compared to west of CTR.

Interestingly, we found a good concordance between the different Bayesian and non-Bayesian methods used in this study. The patchy spatial patterns in RTR and CTR, detected by STRUCTURE and GENELAND, were also supported by DAPC and sPCA analysis. Some ambiguity in the individual assignment in STRUCTURE (with prior and without prior) might be due to the presence of a weak population structure [100]. The principle component differentiated the RTR population (Cluster 4; see S3a Fig) from the other clusters of CTR and its adjoining FDs. The distinct RTR cluster suggested significant differentiation, but a small overlap of cluster 4 of RTR with CTR might indicate moderate gene flow. Presence of a weak population structure detected using the Bayesian methods in CTR and its adjoining FDs indicate sufficient genetic exchange between these areas. Separate DAPC analysis with CTR and adjoining FDs (S3b Fig), indicated that the subdivision in CTR and its adjoining FDs is not very strong. Hence, the weak genetic structure in the habitats of eastern CTR may be attributed to a low level of disturbance (Table 5). GLSI analysis also showed sharper “ridges” in CTR and the adjoining FDs to the east, indicating that the greatest genetic distances are between CTR and RTR (Fig 3).

Detection of first-generation migrants (GENECLASS 2) and asymmetric migration in the last three to five generations (BAYESASS) between CTR and RTR provides evidence of migration between the populations even with this level of disturbance. Two migrants and one admixed individual were detected between RTR and CTR, which suggests that the Rajaji–Corbett corridor is functional but at a low scale. BAYESASS identified contemporary migration and suggested that there is asymmetric migration from CTR to RTR, with a rate of more than 5% (m = 0.089). This asymmetric gene flow is characteristic for source–sink population dynamics, with individuals moving from more stable, higher density populations (CTR) into a neighboring low-density population (RTR). Emigrant tigers from CTR to eastern RTR are probably exploring areas of low disturbance in order to settle there. Harihar et al. [37] reported a high turnover of tigers in eastern RTR, determined through camera traps. This high turnover is probably the result of tigers coming from CTR, as this is the only source population in this landscape. At the same time, the high migration from CTR maintains demographic connectivity among the populations. Harihar and Pandav [39] indicated that the eastern RTR (Chilla) has a fairly good prey density, but disturbance-free areas are very limited and already have resident tigers. Of the 10 transects surveyed in this area in 2008, only two were disturbance-free, whereas the livestock and human encounter rates were between 25 and 100/km in remaining transects [39]. Studies on tigers in other landscapes have also indicated that the presence/occupancy of tigers is relatively high in disturbance-free areas [89]. Both the significant genetic differentiation in some areas and the presence of a weak population structure in other regions in WTAL can be supported and explained by the human influence, i.e. human settlement history due to the malaria eradication program and recent anthropogenic and development activities in this tiger landscape.

### 4.3. Human influence

In Asia, the TAL region is one of the most threatened and fragmented landscapes [101], and the history of human habitation can be traced to ancient times. In the early 1950s, the TAL was used only by native tribes because of the prevalence of malaria, but after successful eradication of malaria in the 1960s, migrants started entering the terai from different parts of India. The settlers cleared the TAL forest and used the land for agriculture, as a result of which now only 2% of the natural habitat in this landscape is contiguous [101]. With the change in the land use pattern, the forest areas became fragmented patches, especially in the plains [102]. In addition, the increased human population in this landscape exerts a variety of pressures on forest resources, i.e. extracting wood for fuel, collecting fodder and grazing livestock.

**Fig 5:**
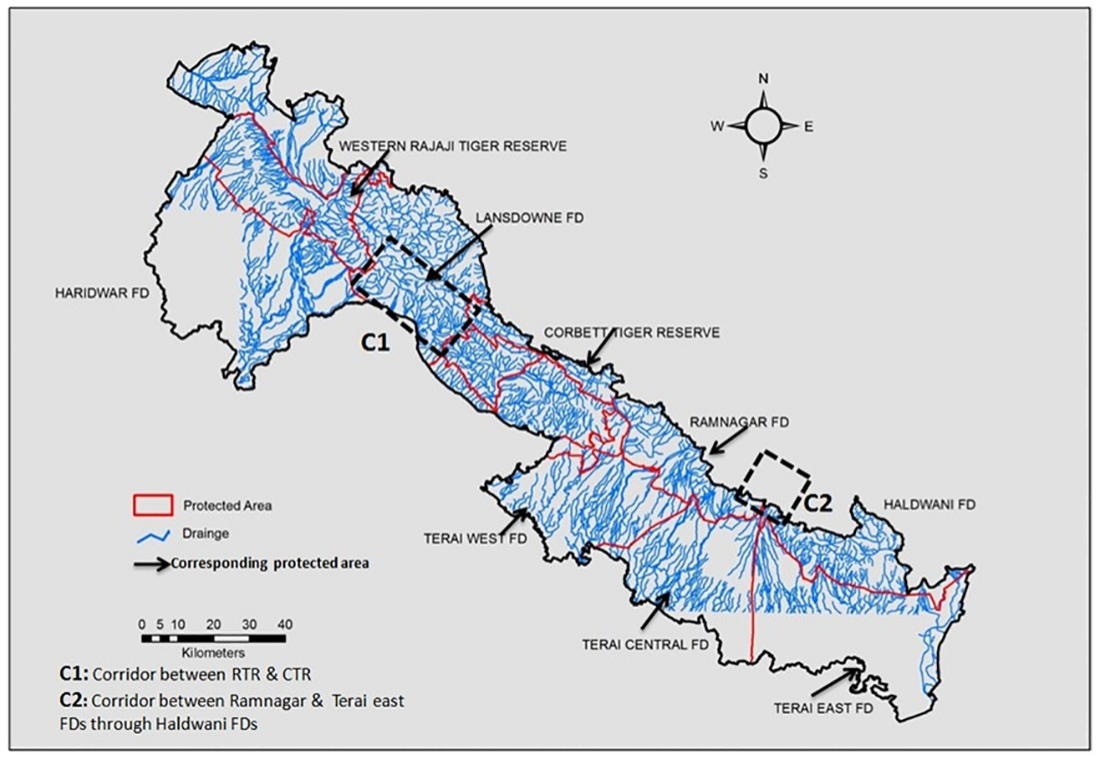
Intensity and spatial distribution of “rivulet” network in WTAL, India.

The increasing human population and its demands and various development-related infrastructure projects have broken the connectivity of the forest in this tiger landscape, as evidenced in night light intensity (Fig 1). Various development activities in the form of industrial setups and the road and rail networks in WTAL started in the 1960s [102]. The natural mixed forest and grassland in Haridwar, Bijnore, Terai West, Terai Central and Terai East FDs (Fig 1) have been converted into monoculture plantations (such as *Eucalyptus* spp., *Ailanthus excelsa*, *Populus ciliate*) to meet industrial needs. Johnsingh and Negi [34] reported that the extinction of the tiger from the Bijnore FD was due to the conversion of natural forest (which was home for prey species, such as the sambar and wild pig) to monoculture plantations and related human disturbances. During the survey of the Rajaji–Corbett corridor (250 km^2^) in 2000, the authors recorded only three pugmarks of tigers and emphasized the seriousness of the anthropogenic pressure in some parts of this landscape. Joshi et al. [103] also reported that in the last decade, movements of wild elephants and tigers have been rare due to the heavy vehicle traffic on the Kotdwar–Lansdowne and Kotdwar–Kalagarh highways (Fig 1). The township and agricultural lands in Kotdwar severely threaten both of the corridors connecting Rajaji to Corbett, especially Bijnore [88]. Recent studies [38, 39] also confirmed that the loss of functionality of the regional corridor has resulted in a decrease in tiger occupancy in eastern RTR and suggested maintaining the Rajaji–Corbett corridor by eliminating disturbance and facilitating movement between the populations. This area is relatively flat and has fewer networks of “drainage” or “rivulets” than other areas of this landscape (east of CTR) (Fig 5) and has always been more prone to human encroachment than mountainous terrains. Such “drainage” or “rivulets” in mountainous terrains provide secluded places to move from one area to another. This could have been one of the reasons for the higher gene flow towards the east than the west of CTR.

Several ecological studies carried out in this landscape [32, 36, 38 39] have highlighted the anthropogenic pressure in all connecting corridors in WTAL and have recommended preservation of the Rajaji–Corbett corridor to sustain the tiger population in RTR. Subsequent to the relocation of Gujjars from eastern Rajaji (Chilla Range), Harihar et al. [37, 38] reported the recovery of three to four tigers in the eastern part and provided a photo of a lactating female with cubs. This recovery was attributed to the movement of tigers from the source population (CTR) through the Rajaji–Corbett corridor. Our finding of the sibship relationship between Rajaji and Corbett also indicates movement between the two populations. Though there is no direct genetic confirmation of migration of particular individuals from Corbett to Rajaji, the detection of first-generation migrants, considerable asymmetric migration, and sibship relationships strengthen the proof of the functionality of the corridor and the need to minimize disturbance towards the west of CTR to establish another source population. Based on prey abundance, Harihar and Pandav [39] estimated that eastern RTR (Chilla) be able to sustain between 11 and 15 tigers. The high gene flow and weak population structure between CTR and adjoining FDs (Ramnagar FD, Terai west and Central FDs) support the recommendations of Johnsingh and Negi [34] for a “Greater Corbett” from the east of the Rajai–Corbett corridor to the Boar River (Fig 1). The priority should be to monitor this “Greater Corbett” area at least annually with reference to the extent of human disturbance so that timely management initiatives may be taken up to increase the dispersal of tigers across populations.

## 5. Conservation implications

Our results have important implications for the management of the tigers in the WTAL region.

1. Our data suggest the presence of low to moderate gene flow among different populations in WTAL. Therefore, we suggest immediate conservation strategies to minimize anthropogenic factors especially in the two identified corridors, i.e., C1 and C2 (Fig 1). Movement of tigers between populations may be aided by the extensive drainage or rivulet network, which is known to facilitate such movements in presence of low to moderate disturbance (Fig 5). The absence of genetic structuring towards the east in CTR reveals a need for continuous monitoring of the habitat quality and urges action to minimize anthropogenic pressure. This is similar to the suggestion of Johnsingh and Negi [34] that the area from the east of the Rajaji–Corbett corridor to the Boar River should be treated as the “Greater Corbett” Conservation Area.
2. The GLSI, GENELAND and allele sorting analyses towards the east in Corbett between Ramnagar and Terai east FDs (Fig 3) around corridor C2 along the Gola river (Fig 1) indicate less gene flow among the resident populations. It is recommended that until the suggestions regarding sand mining and boulder collection are implemented [104], the quality of the forests north of Haldwani along the Gola river (C2) (Fig 1) should be retained and the anthropogenic factors and development in these forest patches should be minimized. This may enable the tigers to move from Ramnagar FD to Terai East FD through the Haldwani FD (Fig 1), which may minimize further population differentiation in Terai East FD. The same corridor has also been identified by Qureshi et al. [88].
3. As tiger are philopatric, related individual are expected in a stable population. However the observed distinct genetic clusters (both Bayesian and non-Bayesian), significant genetic differentiation, and low sibship relationship between CTR and RTR reveal the absence of a viable stable population in the eastern RTR (Chilla). This may also be supported by the presence of high turnover of the individuals observed during camera trapping, though the area could sustain between 11 and 15 tigers [39]. This may be due to intense anthropogenic activities and a limited disturbance-free area. Stable population has been reported from disturbance free or inviolate areas; therefore, conservation efforts should be aimed at minimizing the level of disturbance in C1 between these two areas (Fig 1).
4. Besides habitat fragmentation, poaching has been a major threat in this landscape because there are large numbers of villages in the plains of southern TAL. In a habitat that follows a “stepping-stone” pattern, emigrants have higher probability of coming in contact with human habitation while dispersing. Therefore, the observed genetic diversity and viability of this source population in CTR may be maintained through high reproductive success and by providing adequate protection in the adjoining areas so that floater males are able to contribute to the gene pool, which will reduce inbreeding.
5. To maintain connectivity and avoid human–wildlife conflicts in this landscape, the measures that have been suggested previously [34, 37, 39, 47, 48] relocation of villages and industries, prevention of encroachment, and banning sand and boulder mining in the corridor should be implemented. All these measures will facilitate dispersal of tigers from CTR to RTR. With the recent declaration of the Rajaji National Park to the present Rajaji Tiger Reserve (RTR), management may improve and facilitate conservation even to an extent that there will be another source population in WTAL.
6. Monitoring of genetic characteristics across this landscape at least once every five years is suggested so the appropriate corrective measures could be taken in case the level of genetic structuring increases.

## Supporting information

S1 Table Genotyping error rates (ADO = Allelic Dropout, FA = False Allele) at 13 microsatellite loci with n = 7 scat samples for RTR. (DOCX)

S2 Table AMOVA variations within and between tiger populations of WTAL, India. (DOCX)

S3 Table Summary of migrant assignments made on the basis of GENECLASS analysis. (DOCX)

S1 Fig STUCTURE plots for K = 2 and 3, based on 10 microsatellite loci, admixture model, with prior information of the geographical origin of the samples. (DOCX)

S2 Fig Selection of best possible number of genetic clusters on the basis of DIC criterion for BYM detecting two genetic populations. Individual assignment probabilities of Bengal tiger to genetic clusters using the model-based program TESS run of *K* = 2.
(DOCX)

S3 Fig Results of DAPC analysis: Each population is shown in an ellipse of a different color. (a) Results with RTR, CTR and adjoining forest divisions; (b) results with CTR and adjoining forest divisions. In the bar plots of both figures each individual is represented by a single vertical colored line and lengths of colored line is proportional to each of the inferred clusters.
(DOCX)

S4 Fig Interpolation using a globally weighted regression of component 1 scores from sPCA. Contours are component scores representing similarity across the landscape.
(DOCX)
