# Supplementary material for: Fine-scale population genetic structure of the Bengal tiger (Panthera tigris tigris) in a human-dominated western Terai Arc Landscape, India
Source: PLoS One. 2017 Apr 26;12(4):e0174371. doi: 10.1371/journal.pone.0174371 (PMC5405937; doi:10.1371/journal.pone.0174371)
Supplement: S1 Table — (DOCX) [file pone.0174371.s001.docx]

Table S1: Genotyping error rates (ADO = allelic dropout, FA= False allele) at 13 microsatellite loci with n=7 scat samples for RTR.

| Loci name | No of scat samples | ε_1_  (ADO) | ε_2_  (FA) |
| --- | --- | --- | --- |
| PttA2 | 7 | 0.000 | 0.000 |
| PttE5 | 7 | 0.076 | 0.011 |
| PttF4 | 7 | 0.101 | 0.000 |
| PttD5 | 7 | 0.157 | 0.000 |
| PUN100 | 7 | 0.000 | 0.000 |
| PUN327 | 7 | 0.178 | 0.000 |
| FCA304 | 7 | 0.096 | 0.000 |
| Fca272 | 7 | 0.000 | 0.000 |
| F41 | 7 | 0.000 | 0.050 |
| FCA126 | 7 | 0.000 | 0.000 |
| FCA672 | 7 | 0.00 | 0.000 |
| FCA232 | 7 | 0.000 | 0.000 |
| Fca090 | 7 | 0.140 | 0.000 |
