# Supplementary material for: Fine-scale population genetic structure of the Bengal tiger (Panthera tigris tigris) in a human-dominated western Terai Arc Landscape, India
Source: PLoS One. 2017 Apr 26;12(4):e0174371. doi: 10.1371/journal.pone.0174371 (PMC5405937; doi:10.1371/journal.pone.0174371)
Supplement: S2 Table — (DOCX) [file pone.0174371.s002.docx]

Table S1: AMOVA variations within and between tiger populations of WTAL, India.

| Source of variation | d.f | Sum of squire | Variance component | Percentage of variation |
| --- | --- | --- | --- | --- |
| Among group | 2 | 11.00 | 0.0763 Va | 4.54 |
| Among population within group | 1 | 2.59 | 0.262 Vb | 1.56 |
| Within Populations | 138 | 218.04 | 1.58 Vc | 93.90 |
| total | 141 | 231.634 | 1.72120 |  |
| Fixation Index | *F_ST_=0.061 (p<0.001)*  *F_CT_=0.022 (p=0.011)*  *F_SC_=0.039 (p=0.022)* | | | |
