# Supplementary material for: Fine-scale population genetic structure of the Bengal tiger (Panthera tigris tigris) in a human-dominated western Terai Arc Landscape, India
Source: PLoS One. 2017 Apr 26;12(4):e0174371. doi: 10.1371/journal.pone.0174371 (PMC5405937; doi:10.1371/journal.pone.0174371)
Supplement: S3 Table — (DOCX) [file pone.0174371.s003.docx]

Table S3: Summary of migrant assignments made on the basis of GENECLASS analysis.

| Serial number | SID | Sex | Sampled locality | GENECLASS2  assigned Pop | GENECLASS2  probability (Lh/Lmax) |
| --- | --- | --- | --- | --- | --- |
| 1 | 1081 | Female | CTR | RTR | 2.486 |
| 2 | 7748 | Male | RTR | CTR | 1.014 |
| 3 | 209 | Male | RTR | CTR | 3.681 |
