# Supplementary figures and images for: Fine-scale population genetic structure of the Bengal tiger (Panthera tigris tigris) in a human-dominated western Terai Arc Landscape, India
Source: PLoS One. 2017 Apr 26;12(4):e0174371. doi: 10.1371/journal.pone.0174371 (PMC5405937; doi:10.1371/journal.pone.0174371)

Figure S1


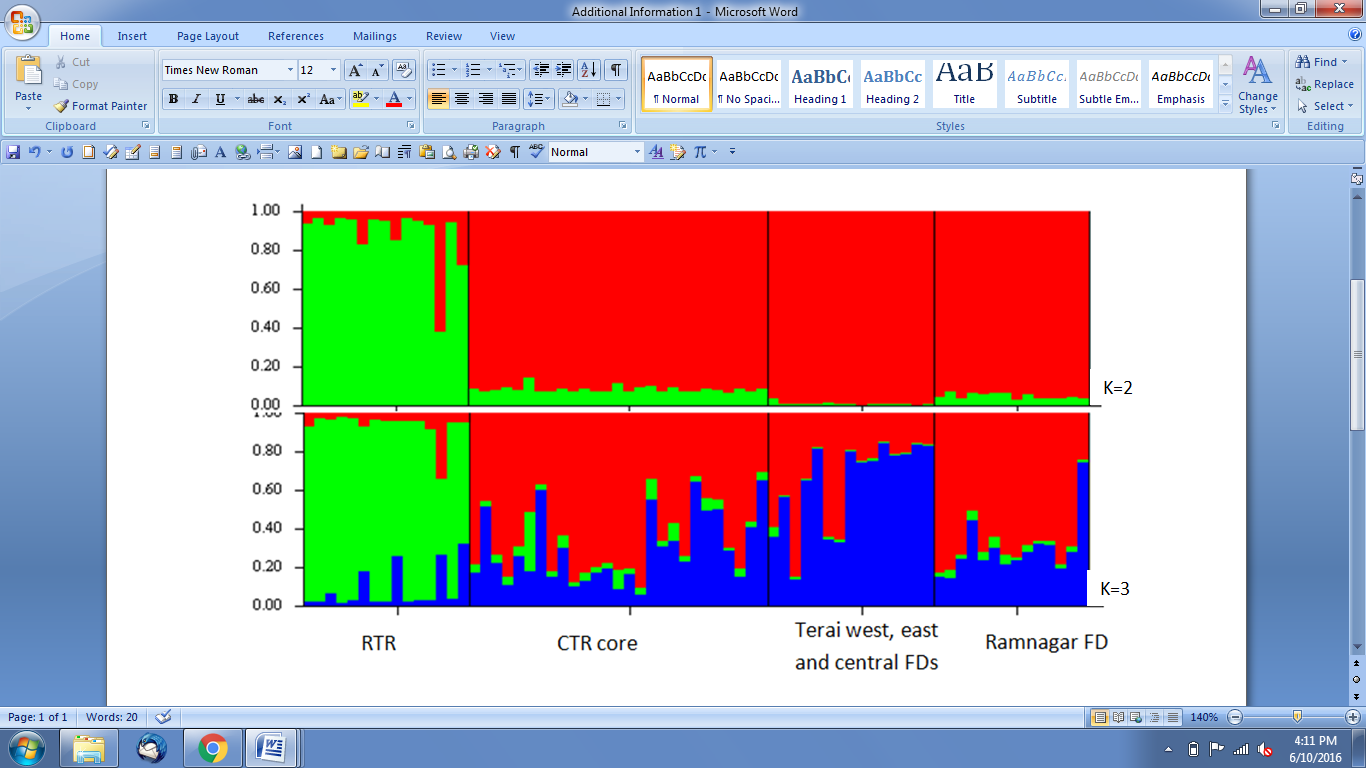

Supplement: S1 Fig — (DOCX) [file pone.0174371.s004.docx]

Figure S2


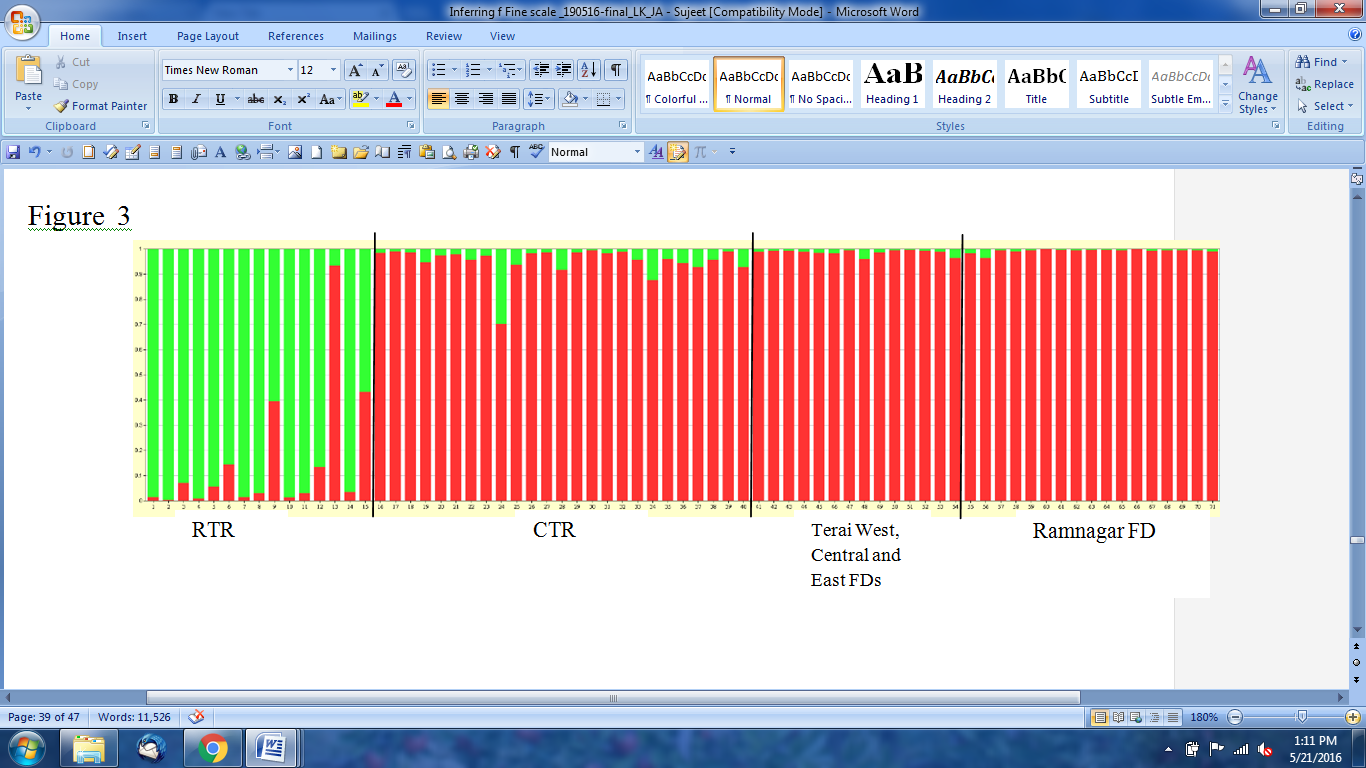

Supplement: S2 Fig — Individual assignment probabilities of Bengal tiger to genetic clusters using the model-based program TESS run of K = 2. (DOCX) [file pone.0174371.s005.docx]

Figure S3


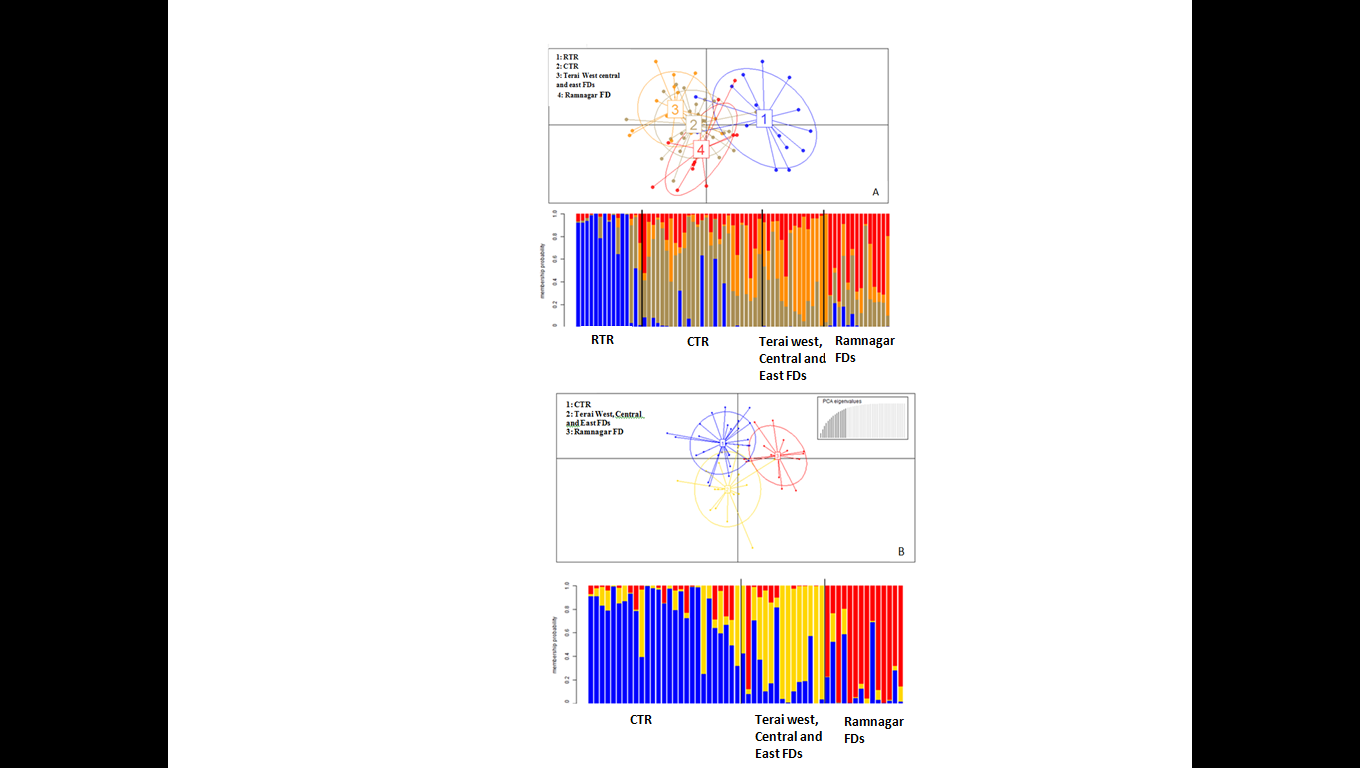

Supplement: S3 Fig — (a) Results with RTR, CTR and adjoining forest divisions; (b) results with CTR and adjoining forest divisions. In the bar plots of both figures each individual is represented by a single vertical colored line and lengths of colored line is proportional to each of the inferred clusters. (DOCX) [file pone.0174371.s006.docx]

Figure S4


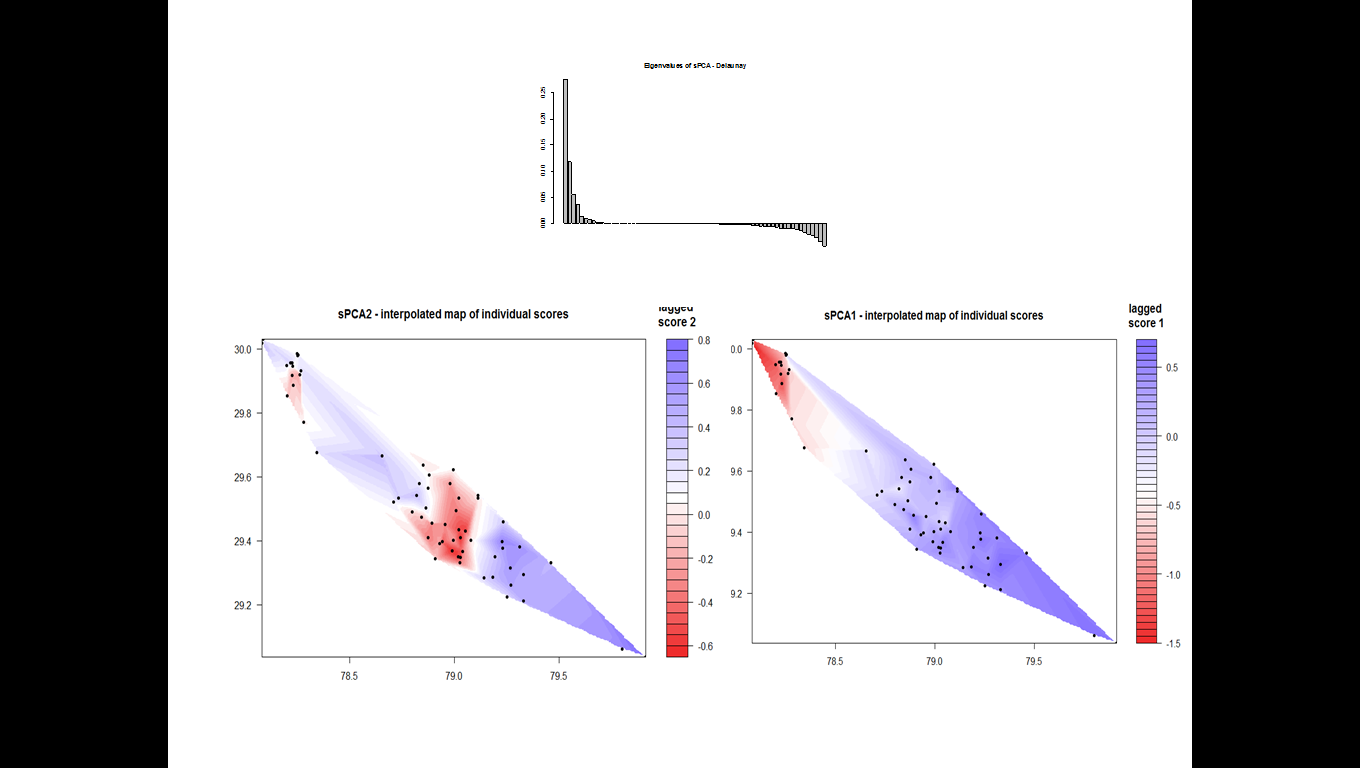

Supplement: S4 Fig — Contours are component scores representing similarity across the landscape. (DOCX) [file pone.0174371.s007.docx]
